# Side Information Generation Scheme Based on Coefficient Matrix Improvement Model in Transform Domain Distributed Video Coding

**DOI:** 10.3390/e22121427

**Published:** 2020-12-17

**Authors:** Wei Wang, Jianhua Chen

**Affiliations:** School of Information Science and Engineering, Yunnan University, Kunming 650000, China; weiwang@mail.ynu.edu.cn

**Keywords:** distributed video coding, side information, Wyner–Ziv frame, arithmetic coding, coefficient matrix improvement model

## Abstract

In order to effectively improve the quality of side information in distributed video coding, we propose a side information generation scheme based on a coefficient matrix improvement model. The discrete cosine transform coefficient bands of the Wyner–Ziv frame at the encoder side are divided into entropy coding coefficient bands and distributed video coding coefficient bands, and then the coefficients of entropy coding coefficient bands are sampled, which are divided into sampled coefficients and unsampled coefficients. For sampled coefficients, an adaptive arithmetic encoder is used for lossless compression. For unsampled coefficients and the coefficients of distributed video coding coefficient bands, the low density parity check accumulate encoder is used to calculate the parity bits, which are stored in the buffer and transmitted in small amount upon decoder request. At the decoder side, the optical flow method is used to generate the initial side information, and the initial side information is improved according to the sampled coefficients by using the coefficient matrix improvement model. The experimental results demonstrate that the proposed side information generation scheme based on the coefficient matrix improvement model can effectively improve the quality of side information, and the quality of the generated side information is improved by about 0.2–0.4 dB, thereby improving the overall performance of the distributed video coding system.

## 1. Introduction

Traditional video coding standards [1,2], such as H.264 [3] and MPEG, all perform complex motion estimation at the encoder side in order to obtain higher video quality while maintaining higher compression performance. This kind of encoding architecture makes the computational complexity of the encoder far higher than that of the decoder, which is more suitable for application scenarios where encoding once and decoding multiple times, such as digital TV and DVD playback and other video services. However, with the popularization and development of wireless low-energy video sensor networks, wireless video surveillance systems [4], and handheld mobile video terminal devices, users have put forward new requirements for video coding. Since most of these wireless devices are battery-powered, their energy supply and computing power are very limited, the traditional video coding framework is challenged in terms of the computational complexity of the encoder.

A different video coding architecture, distributed video coding (DVC) [5], has begun to attract the attention of researchers. The main advantage of DVC is that it can reduce the computational burden on the encoder side in the video coding framework while achieving high compression performance, which is more suitable for mobile video devices with limited energy. DVC is based on Slepian–Wolf theory [6] and Wyner–Ziv theory [7], it realizes the efficient compression of video information with the lower computational complexity at the encoder side. In the DVC framework, different video frames at the encoder side can be regarded as different sources, and they can be independently encoded, that is, the encoder ignores the correlation between these sources, while the decoder has to jointly decode them, which means that the decoder is responsible for exploiting the redundant information among different sources.

In order to further improve the performance of DVC, researchers have proposed various schemes. A new technique to realize Slepian–Wolf coding was presented in [8], and it is used for DVC. The authors showed that coding the positions of the symbols, instead of their values, can be a good way to implement efficient Slepian–Wolf coding and can reduce the complexity of both the encoder and the decoder. Based on this idea, they proposed a practical DVC system. In [9], a frame-level DVC system based on the rate control at the encoder was proposed, which effectively improves the rate distortion (RD) performance of the DVC system at low bit rates. In [10], a new side information successive refinement algorithm was proposed, which uses the additional information obtained after the decoding of the previous discrete cosine transform (DCT) bands of a Wyner–Ziv frame to refine the initial side information frame. This algorithm can considerably improve the RD performance of the DVC system. In [11], an algorithm combined with naive Bayesian theory was proposed to create a general model for the generation of side information in DVC. An ensemble of multilayer perceptron networks for side information generation in DVC was proposed in [12], the main goal of this method is to minimize the estimation error between the side information frame and the corresponding Wyner–Ziv frame, so as to improve the overall efficiency of the DVC systems. In [13], the authors proposed an adaptive two-step side information generation method to improve the DVC system by generating better second-step side information. This method uses the down-sampled decoded Wyner–Ziv frames and the decoded coefficients to progressively improve the RD performance during the decoding procedure. In [14], an efficient scalable DVC scheme was proposed for video transmission in wireless video sensor networks. In their scheme, the scalable Wyner–Ziv frame is based on transmission of different wavelet information, while the key frame is based on transmission of different residual information. The proposed scheme significantly contributes to the performance of a DVC system. In [15], researchers proposed a side information generation method for low-delay DVC, in which the side information results generated respectively by the autoregressive model and the traditional extrapolation method are fused based on a probability model to get the final side information. The experiment results show that the proposed autoregressive model can effectively improve the RD performance. In [16], researchers proposed a DVC scheme using interval overlapped arithmetic coding, where the key frames are compressed using traditional video coder while the Wyner–Ziv frames are compressed using distributed arithmetic coding. The proposed scheme is competitive and has a good RD performance. Video coding based on compressive sensing is also one of the implementation schemes of distributed video coding. For example, Evgeny [17] presented a novel efficient and robust JPEG compatible video coding algorithm based on the compressive sensing framework, which is significantly more robust to packet losses compared to conventional codecs. In [18], researchers presented a compressive-sensing-based video codec with a low-complexity encoder, which is suitable for wireless video system requiring simple encoders but tolerant, more complex decoders. The experiment results demonstrate that the RD performance of the proposed codec is superior to the state-of-the-art compressive-sensing-based video codec.

It is worth noting that the side information generation at the decoder side is an important part of the DVC framework, and the quality of the side information directly affects the performance of Wyner–Ziv frame decoding. Therefore, improving the quality of side information can improve the overall performance of the DVC system. In this paper, we propose an improved side information generation scheme in the transform domain. Firstly, the optical flow method in [19] is used to generate the initial side information. Since the optical flow method can perform motion estimation better, the generation of side information by using the optical flow method is also more advantageous. However, using only key frames to generate the side information for the Wyner–Ziv frame still has some limitations. Secondly, we consider transmitting part of the important information of the Wyner–Ziv frames to the decoder side losslessly, and using this important information to further improve the initial side information, specifically, performing the block-based 4 × 4 DCT [20] for Wyner–Ziv frame at the encoder side, and then the block-based 4 × 4 DCT coefficients are organized into 16 bands by zig-zag scan order. The important coefficients information after DCT is encoded by an adaptive arithmetic encoder [21] and transmitted to the decoder, the side information is also transformed by DCT at the decoder side. Therefore, the important coefficients information decoded by the adaptive arithmetic decoder is used to improve the side information, that is to say, the lossless DCT coefficients information obtained at the decoder is used to replace the DCT coefficients information at the corresponding position in the initial side information. Finally, the coefficient matrix improvement model (CMIM) is used for further improvement based on the lossless coefficients information, thereby the reliability and quality of side information will be effectively improved.

## 2. Distributed Video Coding System

**Figure 1:**
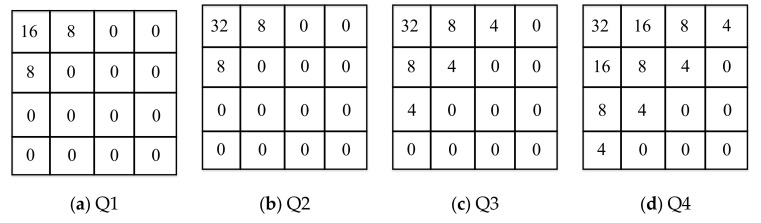

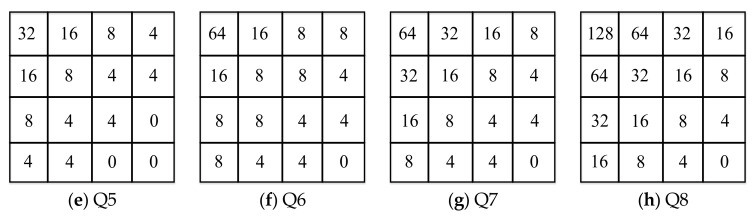
Quantization matrices.

DVC is a video coding scheme that is different from traditional video coding architecture. DVC can reduce the computational complexity of the encoder side while ensuring a high compression ratio. For DVC in the transform domain, the original video sequence is divided into key frames and Wyner–Ziv frames, and for key frames, the traditional video encoder is used to encode them directly, while for Wyner–Ziv frames, a block-based DCT is performed, and then the DCT coefficients of each pixel block in the same position are extracted to form a DCT band. For a Wyner–Ziv frame, if a 4 × 4 block-based DCT is performed, then 16 DCT bands can be obtained. To encode each DCT band, a predefined number of quantization levels are used depending on the quality of the Wyner–Ziv frames [22]. Eight quantization matrices are illustrated in Figure 1. The quantized information is processed by using a low density parity check accumulate (LDPCA) encoder, which can generate the respective syndromes (parity bits) [23]. The larger the number of quantization levels is, the higher the bit rate is, and the higher the decoded video quality will be, so that the decoding results with different bit rates can be obtained. At the decoder side, the key frames are directly decoded by the traditional video decoder, and are used for motion estimation, thereby the side information is obtained. For Wyner–Ziv frames, according to the side information generated at the decoder side and the parity bits transmitted from the encoder, the LDPCA decoder can perform iterative decoding to get the final decoded Wyner–Ziv frames according to the correlation between frames. It can be seen from above that the side information is a noisy version of the Wyner–Ziv frame. The less “noise” of the side information relative to the original Wyner–Ziv frame, the better the final decoded result will be, which means that the quality of the side information will directly affect the decoded result of the Wyner–Ziv frame. Therefore, improving the accuracy of the generated side information is essential for improving the performance of a DVC system.

## 3. DVC System Based on the Proposed Side Information Generation Scheme

**Figure 2:**
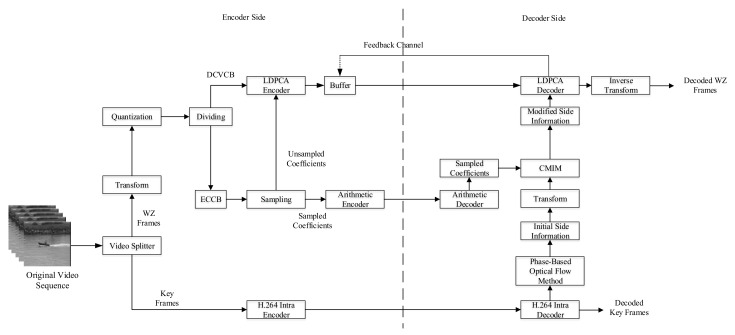
Distributed video coding system based on the proposed side information generation Scheme.

The side information is obtained by motion estimation using the decoded key frames at the decoder side, which is an approximate version of a Wyner–Ziv frame. In order to obtain the ideal side information, the optical flow method [19] is used to generate the initial side information. However, when the coding and decoding parameters of the key frames are constant, especially when the bit rate of the key frame is not high, the generation of side information will still show limitations. In other words, there are many “errors” in the generated side information, which are not conducive to the error correction of the LDPCA decoder. In order to solve such problems, we propose a side information generation scheme based on CMIM. The coefficient bands of the Wyner–Ziv frame after DCT are divided into entropy coding coefficient bands (ECCB) and distributed video coding coefficient bands (DVCCB). The coefficients of ECCB are sampled and divided into sampled coefficients and unsampled coefficients. For the sampled coefficients, an adaptive arithmetic encoder is used for lossless encoding. For unsampled coefficients and the coefficients of DVCCB, the LDPCA encoder is used to calculate the parity bits, and then the lossless sampled coefficients are used with CMIM at the decoder side to improve the coefficient matrix of the side information. As shown in Figure 2, it is a block diagram of DVC system based on the proposed side information generation scheme.

### 3.1. Video Splitter, Transform, and Quantization

It can be seen from the block diagram that the input original video sequence is divided into multiple GOP (group of pictures). When the size of GOP is 2, it means that each group is composed of a key frame and a Wyner–Ziv frame. For the key frames, it needs to be restored with high quality at the decoder side to generate side information, so the H.264 intra encoder is chosen for encoding. For a Wyner–Ziv frame, since the decoder can generate the side information for the Wyner–Ziv frame, we only need to use the LDPCA encoder to generate its parity bits.

The application of DCT is due to the fact that it can remove spatial redundancy of pixels within a frame, which helps improve the performance of DVC.

The two-dimensional 4 × 4 DCT coefficient matrix A can be expressed as:

(1) $$A=C\cdot a\cdot C^{T}$$

where a denotes the 4 × 4 signal matrix and C denotes the DCT transform matrix. C can be expressed as:

(2) $$C=\left[\begin{array}{cc} \begin{array}{cc} b & b\\ h & y \end{array} & \begin{array}{cc} b & b\\ -y & -h \end{array}\\ \begin{array}{cc} b & -b\\ y & -h \end{array} & \begin{array}{cc} -b & b\\ h & -y \end{array} \end{array}\right]=\left[\begin{array}{cc} \begin{array}{cc} 1 & 1\\ 2 & 1 \end{array} & \begin{array}{cc} 1 & 1\\ -1 & -2 \end{array}\\ \begin{array}{cc} 1 & -1\\ 1 & -2 \end{array} & \begin{array}{cc} -1 & 1\\ 2 & -1 \end{array} \end{array}\right]⊗\left[\begin{array}{cc} \begin{array}{cc} b & b\\ \frac{h}{2} & \frac{h}{2} \end{array} & \begin{array}{cc} b & b\\ \frac{h}{2} & \frac{h}{2} \end{array}\\ \begin{array}{cc} b & b\\ \frac{h}{2} & \frac{h}{2} \end{array} & \begin{array}{cc} b & b\\ \frac{h}{2} & \frac{h}{2} \end{array} \end{array}\right]=C_{f}⊗E_{f}$$

where *b* is equal to 1/2, *h* is equal to $\frac{1}{\sqrt{2}}\cos\frac{\pi }{8}$, and *y* is equal to $\frac{1}{\sqrt{2}}\cos\frac{3\pi }{8}$, $C_{f}$ is the integer DCT transform matrix, $E_{f}$ is the correction matrix, and “⊗” denotes the multiplication of the corresponding position elements of the matrices. This integer DCT derives from DCT, preserving original feature of DCT. Its main idea is to separate the floating-point operations in the transform matrix and put them in the quantization stage. Therefore, a DCT-like matrix retains only integer elements for transformation. That means only additions, subtractions, and shifts are used to implement the integer DCT transform. In conclusion, the integer DCT on the signal matrix a can be expressed as:

(3) $$A_{f}={\;C}_{f}\cdot a\cdot C^{T}{}_{f}$$

The DCT based on 4 × 4 blocks will generate 16 DCT coefficient bands, and we rank them by importance according to the zig-zag scanning order.

### 3.2. DCT Coefficient Bands Dividing and Sampling Process

**Figure 3:**
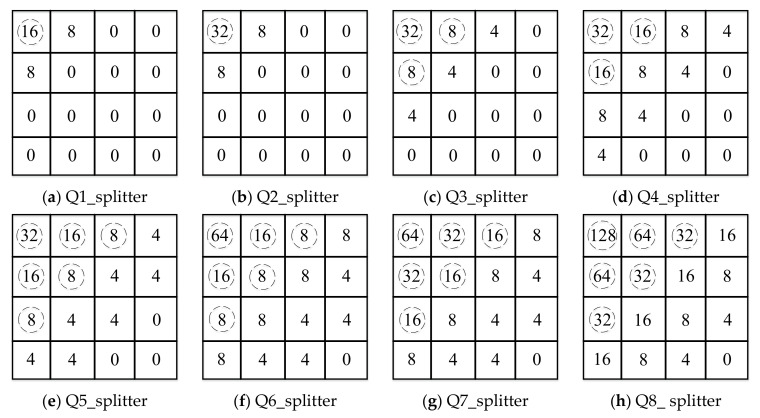
Quantization splitter matrices (Dotted circles indicate the position of the entropy coding coefficients).

**Figure 4:**
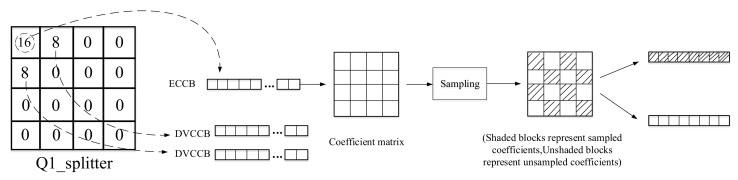
Coefficient bands dividing and sampling process.

For DCT coefficient bands, quantization is also required, and a uniform quantizer with $2^{M_{k}}$ quantization levels is used for quantization, where $2^{M_{k}}\in \left\{0,2,4,8,16,32,64,128\right\}$, $2^{M_{k}}=0$ indicates that the corresponding band do not need to be encoded and transmitted to the decoder, but directly replaced with the corresponding transform coefficient of the side information. Figure 1 shows the 8 quantization matrices Qi (i = 1,2,3,...,8), it is easy to know that the larger the value of i, the higher the bit rate that needed for transmitting, and the higher the quality of the decoded Wyner–Ziv frames. In order to improve the quality of side information, we divide the DCT coefficient bands based on the importance of the quantization matrix and the DCT coefficient bands. The quantization splitter matrices are shown in Figure 3. The specific dividing process is shown in Figure 4 with the quantization splitter matrix Q1_splitter as an example. As can be seen from the figure, for the ECCB, we need to form it into a coefficient matrix, and then sample it according to the way in the figure (the odd-numbered positions of the odd-numbered rows are sampled, and the even-numbered positions of the even-numbered rows are also sampled), so that we can get the sampled coefficients and the unsampled coefficients. For the sampled coefficients, we use an adaptive arithmetic encoder for encoding, and for the unsampled coefficients, we use an LDPCA encoder to calculate the parity bits. In this way, we can use both interframe correlation and intracoefficient correlation at the decoder side, which can effectively improve decoding performance.

### 3.3. Coefficient Matrix Improvement Model (CMIM)

**Figure 5:**
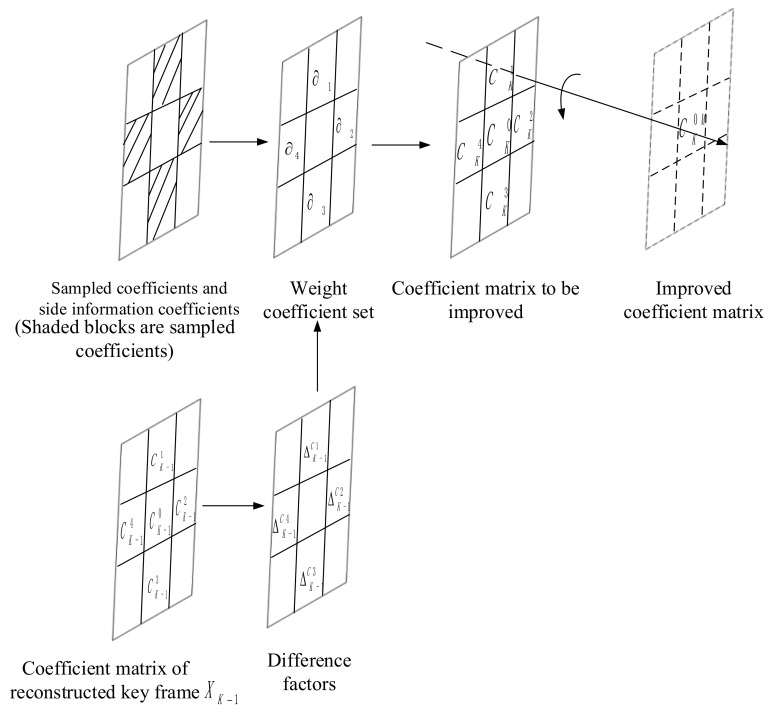
Coefficient matrix improvement model (CMIM).

For the side information generation part, we use the optical flow method in [19] to perform motion estimation to obtain the side information. Since the optical flow method can generate smoother and more accurate motion vectors, the accuracy of the generated side information is also higher. In order to further improve the initial side information generated by the optical flow method, we propose CMIM to improve the initial side information. Specifically, the side information obtained by the decoder side is subjected to a 4 × 4 integer DCT, and the coefficients of the corresponding positions of the initial side information after 4 × 4 integer DCT are extracted to form 16 coefficient matrixes. According to the division of DCT coefficient bands and sampling process, the coefficients in the corresponding coefficient matrix of side information are replaced by the sampled coefficients. In this way, the coefficient matrixes to be improved can be obtained. It is easy to know that this process can effectively improve the quality of side information. However, it should be noted that the above operation is at the cost of requiring more bits. In order to make full use of this part of the sampled information at the decoder side (that is, make full use of the intracoefficient correlation), we modify the coefficient matrices to be improved, mainly using the undistorted sampled coefficients to correct the inaccurate coefficients in the matrices. The variance of each 3 × 3 matrix in the corresponding coefficient matrix of the previous key frame is calculated, thus the average variance $\sigma_{m}^{2}$ of the whole coefficient matrix of the previous key frame can be obtained. The average variance $\sigma_{m}^{2}$ is used as the benchmark to classify each 3 × 3 matrix. Specifically, if the variance of the 3 × 3 matrix in the previous key frame is less than the average variance $\sigma_{m}^{2}$, it means that the texture complexity of this block is low. Assuming that there is a high correlation between adjacent frames, the matrix of the corresponding position of the current side information coefficient matrix 3 × 3 will also show the same texture complexity characteristics. Then the coefficients in the coefficient matrix of the side information generated by the optical flow method will be accurate, that is to say, these coefficients are accurate coefficients. On the contrary, if the variance of the 3 × 3 matrix in the previous key frame is greater than the average variance $\sigma_{m}^{2}$, optical flow method cannot accurately perform a motion estimation. It means that the coefficients in the current side information coefficient matrix are inaccurate coefficients. In this case, we use CMIM to modify them. As shown in Figure 5, suppose any coefficients to be corrected in the coefficient matrix of the initial side information is $C_{K}^{0}$, and its true value is set as $C_{K}^{0R}$. In the coefficient matrix to be improved, we use the adjacent sampled coefficients around the inaccurate coefficient $C_{K}^{0}$ to perform linear weighting and get the coefficient $C_{K}^{0M}$ according to the probability fusion method [24], and $C_{K}^{0M}$ is considered to be an improved version of $C_{K}^{0}$. The adjacent coefficients are set to $C_{K}^{i}\left(i=1,\ldots ,4\right)$.

In the coefficient matrix to be improved, the differences between the real value $C_{K}^{0R}$ and the adjacent coefficients around it are: $C_{K}^{i}-C_{K}^{0R}={∆}_{K}^{i}\;\left(i=1,\ldots 4\right)$. It is impossible to know the true value $C_{K}^{0R}$ of the inaccurate coefficient in the coefficient matrix to be modified, so the real difference ${∆}_{K}^{i}$ could not be obtained. However, the above difference can be estimated indirectly through the decoded key frame $X_{K-1}$. According to the position of the inaccurate coefficient in the current coefficient matrix to be improved, the coefficient at the corresponding position of the key frame $X_{K-1}$ can be located. Additionally, according to the formula: $C_{K-1}^{i}-\;C_{K-1}^{0}=\;{∆}_{K-1}^{Ci}\;\left(i=1,\ldots 4\right)$, the corresponding coefficient differences between the coefficient of the previous key frame $X_{K-1}$ and its surrounding adjacent coefficients can be calculated: ${∆}_{K-1}^{Ci}\left(i=1,\ldots ,4\right).$ It is assumed that the corresponding region between adjacent frames has a strong correlation, that is, the change at the corresponding position of the coefficient matrix between adjacent frames is similar, so we can get equation: ${∆}_{K}^{i}\propto {∆}_{K-1}^{Ci}\;\left(i=1,\ldots 4\right)$. In this way, the difference factors ${∆}_{K}^{i}$ around an inaccurate coefficient in the current side information frame can be estimated by the differences at the corresponding position of the key frame $X_{K-1}:\;{∆}_{K-1}^{Ci}$. Suppose $\alpha_{1},\ldots ,\alpha_{N}$ are the weighting coefficients of the sampled coefficients corresponding to each difference factor, $C_{K}^{1},\ldots ,C_{K}^{N}$ represent the N sampled coefficients around the current inaccurate coefficient, then the corresponding probability fusion result can be obtained according to these weighting coefficients:

(4) $$f\left(C_{K}^{1},\ldots ,C_{K}^{N}\right)=\sum_{n=1}^{N}\alpha_{n}C_{K}^{n}$$

According to Bayesian rules:

(5) $$\alpha_{n}=p\left(n|f\left(C_{K}^{1},\ldots ,C_{K}^{N}\right)\right)$$

The *a posteriori* probability can be obtained by (6):

(6) $$p\left(n|f\left(V_{K}^{1},\ldots ,V_{K}^{N}\right)\right)=\frac{p\left(f\left(V_{K}^{1}\ldots ,V_{K}^{N}\right)|n\right)p\left(n\right)}{\sum_{l=1}^{N}p\left(f\left(V_{K}^{1},\ldots ,V_{K}^{N}\right)|l\right)p\left(l\right)}$$

$p\left(n\right)$ represents the *a priori* probability of the nth sampled coefficient. Apparently, $p\left(n\right)=1/N$. Suppose $p(f\left(C_{K}^{1}\ldots ,C_{K}^{N}\right)|n)$ is a Gaussian probability function:

(7) $$p\left(f\left(C_{K}^{1}\ldots ,C_{K}^{N}\right)|n\right)=p\;\left({∆}_{K}^{n}\right)\propto \exp\left(-{∆}_{K}^{n}{}^{2}\right)$$

By replacing $p\left(f\left(C_{K}^{1}\ldots ,C_{K}^{N}\right)|n\right)$ in (6) with (7) and considering (5), we have:

(8) $$\alpha_{n}=p\left(n|f\left(C_{K}^{1},\ldots ,C_{K}^{N}\right)\right)=\frac{\exp(\frac{-{∆}_{K}^{n}{}^{2}}{2\sigma_{w}{}^{2}})}{\sum_{l=1}^{N}\exp(\frac{-{∆}_{K}^{l}{}^{2}}{2\sigma_{w}{}^{2}})}$$

The parameter $\sigma_{w}{}^{2}$ in the formula can be used to adjust the shape of the Gaussian probability distribution function, which is empirically set to 50.

By using CMIM, the side information coefficient matrices to be improved can be further modified, thereby improved side information can be obtained.

## 4. Experiment Results and Analysis

In this section, we conduct a lot of experiments to demonstrate the effectiveness of the proposed scheme. Key frames are encoded with H.264/AVC intra. The video sequences used in this experiment are standard video test sequences (QCIF@15Hz): Coastguard, Soccer, Hall Monitor, Foreman. We conduct experiments on the standard test sequence to evaluate the quality of side information (the evaluation standard is Peak-Signal-to-Noise Ratio (PSNR)).

**Figure 6:**
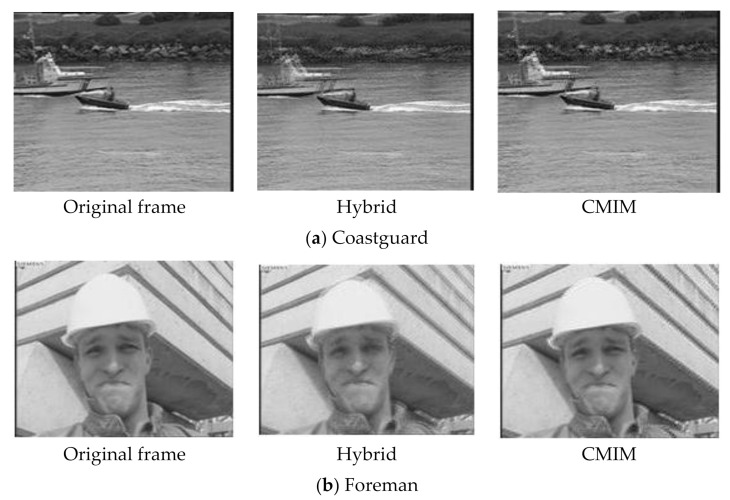
Comparison of subjective quality of side information.

We compare the quality of side information generated by each scheme in Table 1. They are extra [25], OF [26], optical flow [19], hybrid (Qi, i = 1, replace the coefficients in the side information coefficient matrix with sampled coefficients, without CMIM), and the proposed method (CMIM). The quantization parameter QPs are chosen as in [26]. The GOP size in this experiment is 2. It can be seen from Table 1 that the proposed method can generate higher quality side information than other schemes. For the Coastguard sequence, the side information generated by the proposed scheme is 4.96, 1.74, and 3.49 dB higher than that of extra [25], OF [26], and optical flow [19], respectively. For the soccer sequence, the side information generated by the proposed scheme is 5.03, 0.78, and 2.18 dB higher than that of extra [25], OF [26], and optical flow [19], respectively. It can be seen that for the Soccer sequence, the overall quality of side information is not good. This is due to the fact that there are multiple moving objects in the Soccer sequence and the video motion intensity is high. For the Hall Monitor sequence, because the motion intensity of the whole video is small, the quality of side information generated by each method is relatively high. For the Foreman sequence, the side information generated by the proposed scheme is 7.26, 1.05, and 3.03 dB higher than that of extra [25], OF [26], and optical flow [19], respectively. Besides, the proposed scheme can improve the quality of side information generated by hybrid, and PSNR of the proposed side information generation scheme is about 0.4–0.2 dB higher than that of the hybrid. In particular, for the Hall Monitor sequence, the background of the video is almost static, therefore, the improvement effect of the proposed model is limited. Figure 6 is a comparison of the subjective quality of the generated side information, where the generated side information frames by the hybrid scheme and the proposed CMIM scheme are compared to show the effectiveness of CMIM. Generally speaking, the subjective quality of side information improved by the proposed model is obviously different from that without the model. After the improvement with the proposed model, the ghosting and blocking effects almost disappear completely. For example, in the subjective quality comparison of the Coastguard sequence in Figure 6a, the hull in the video frame improved by CMIM become clearer and the blocking effects are significantly reduced, which is closer to the original frame.

**Figure 7:**
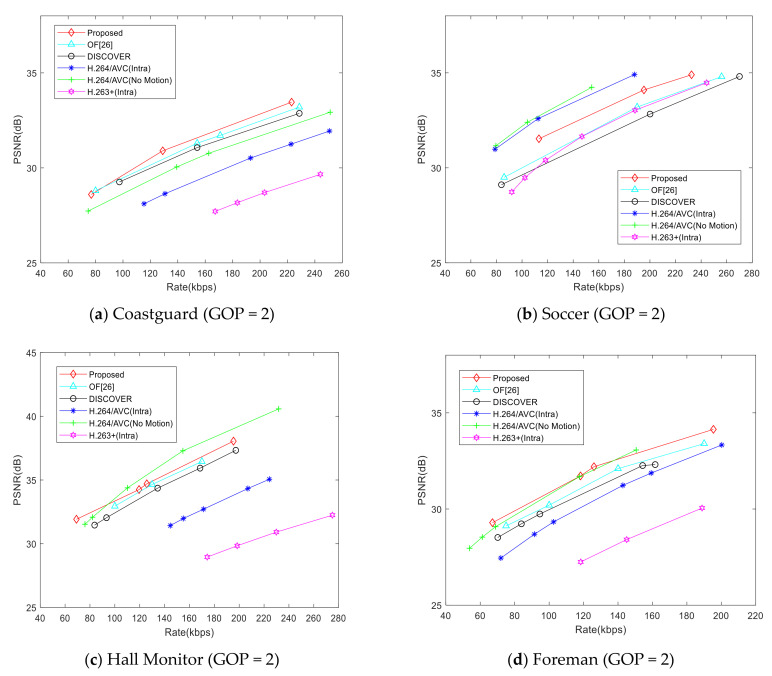
Rate distortion (RD) performance comparison (group of pictures (GOP) = 2).

**Figure 8:**
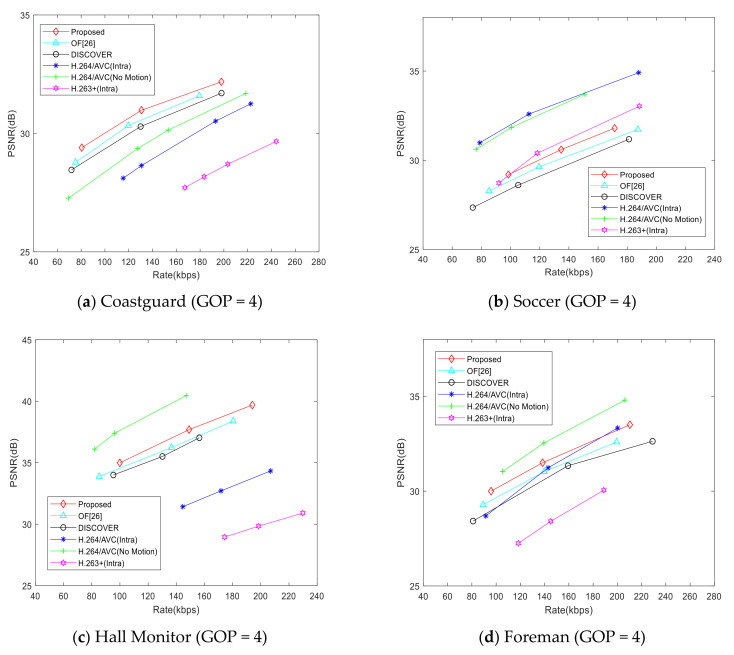
RD performance comparison (GOP = 4).

**Figure 9:**
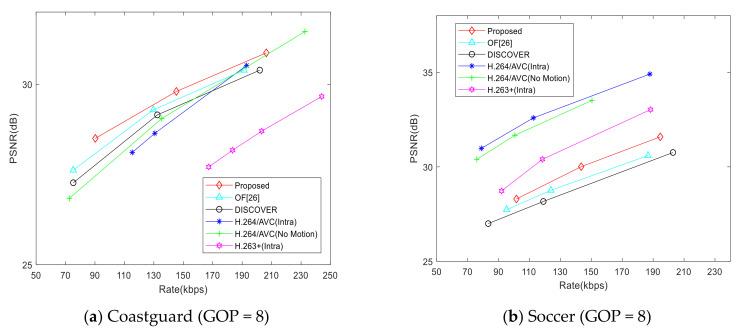

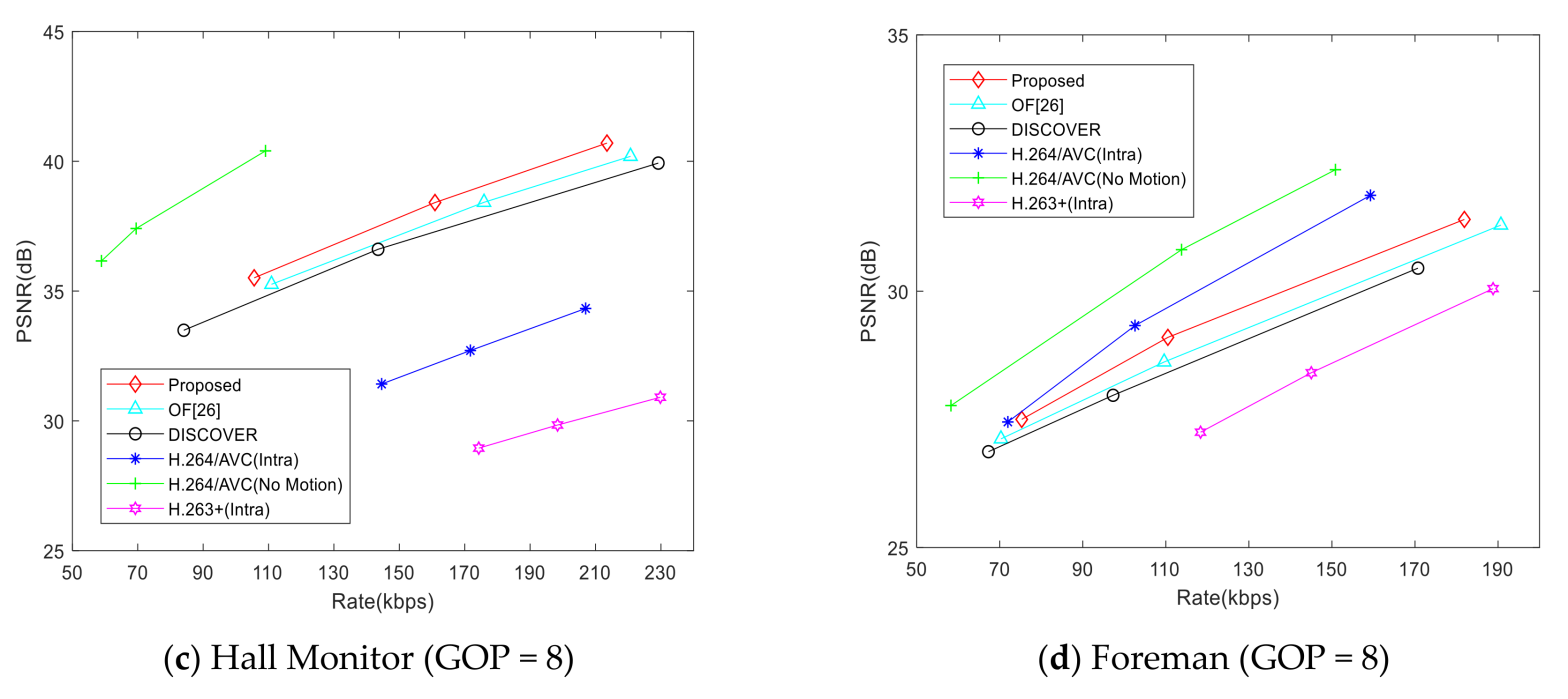
RD performance comparison (GOP = 8).

However, just comparing the side information generation schemes is not enough to reflect the effectiveness of the proposed scheme, we compare the RD performance in Figure 7, Figure 8 and Figure 9 so that the decoding quality can be objectively compared under the same bit rate.

Figure 7 shows the RD performance of each scheme. We compare the proposed scheme with [26], the DISCOVER scheme [22], H.264/AVC (Intra), H.264/AVC (No Motion), and H.263+ (Intra). For the Coastguard test sequence, the RD performance of the proposed scheme is better than that of [26] and the DISCOVER scheme, especially when the bit rate is greater than 80. For the Soccer test sequence, the RD performance of the proposed scheme will gradually exceed that of the hybrid scheme and the DISCOVER scheme, but there is still a big gap between H.264/AVC (Intra) and H.264/AVC (No Motion), which may be caused by the motion intensity and video motion characteristics of the Soccer video sequence. For the Hall Monitor test sequence, the background of the video is almost static, but the motion of characters is not a simple translation, so the RD performance of the proposed scheme is slightly worse than that of H.264/AVC (no motion) when the bit rate is greater than 80, but it is still better than that of [26], DISCOVER, H.264/AVC (intra), and H.263 + (intra). Compared with the DISCOVER scheme, the gain of the proposed scheme is about 0.2–0.6dB. This means that the proposed CMIM scheme can further narrow the gap with H.264/AVC (no motion) in RD performance. For the Foreman test sequence, the motion of objects basically is simple translation. The RD performance of the proposed scheme is better than that of the [26], and the gain is about 0.5dB. To sum up, the proposed CMIM in this paper can effectively improve the side information quality while simultaneously improve the RD performance of the DVC system.

Figure 8 and Figure 9 show the RD performance comparison in the condition of GOP = 4 and GOP = 8. It can be seen from the figures that the RD performance of the proposed scheme is better than that of [26] and the DISCOVER scheme generally. Besides, the RD performance gain of the proposed scheme is also improved compared with that of GOP = 2. However, when the GOP size is increased, the gap between the RD performance of the proposed scheme and H.264/AVC (No Motion) is further widened, especially for the Soccer sequence. This is due to the large motion intensity of the Soccer sequence, which is not conducive to the generation of accurate side information.

**Figure 10:**
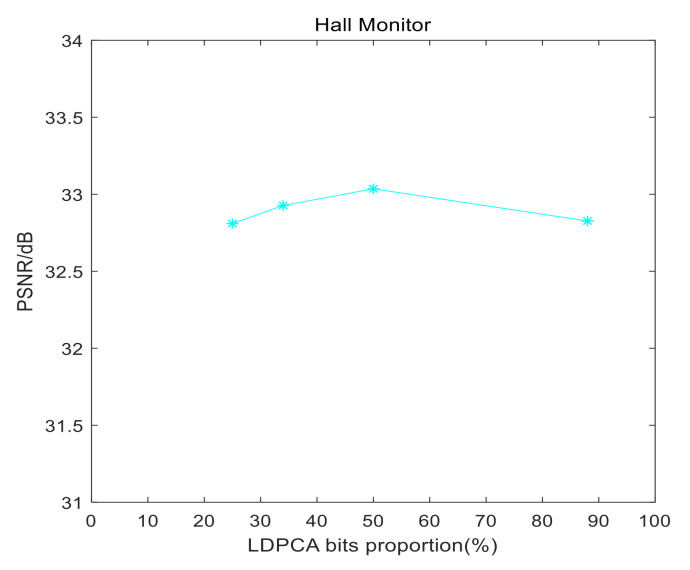
PSNR for different low density parity check accumulate (LDPCA) bits proportion.

It should be pointed out that the rate allocation between LDPCA bits and arithmetic coding bits in this work might not be the best solution. That is to say, there is an optimal balance point between LDPCA bits and arithmetic coding bits to get the best PSNR for a given fixed number of overall bits. Therefore, we take Q1_Splitter as an example, QP = 37, to conduct experiment with the standard test sequence Hall Monitor. As shown in Figure 10, we can see that when LDPCA bits proportion is about 50%, PSNR is the best. It should be noted that this is only for the Hall Monitor sequence, and the best balance point might be different for different test sequences.

## 5. Conclusions

In this paper, a coefficient matrix improvement model is proposed to improve the quality of side information. We divide the DCT coefficient bands of the Wyner–Ziv frame into entropy coding coefficient bands and distributed video coding coefficient bands at the encoder side, in which the coefficients of entropy coding coefficient bands are divided into unsampled coefficients and sampled coefficients. Sampled coefficients are encoded by an adaptive arithmetic encoder, so that it could be restored without distortion at the decoder side. Unsampled coefficients and the coefficients of distributed video coding coefficient bands are encoded by the LDPCA encoder to obtain parity bits. At the decoder side, the optical flow method is used to generate the initial side information. Besides, the decoded lossless sampled coefficients are used to further improve the initial side information with the coefficient matrix improvement model, so as to obtain higher quality side information. Experiment results show that the proposed scheme can effectively improve the quality of side information, and in terms of RD performance, the proposed scheme is generally better than [26] and the DISCOVER scheme.

In future research, we will try to find the best rate balance between the LDPCA encoder and arithmetic encoder and improve the sampling process to further improve the rate distortion performance of distributed video coding.

## Figures and Tables

**Table 1:** Average PSNR comparison of generated side information (Qi, i = 1).

Sequences	Extra [25]	OF [26]	Optical Flow [19]	Hybrid	CMIM
Coastguard	28.55 dB	31.77dB	30.02 dB	33.08 dB	33.51 dB
Soccer	19.26 dB	23.51 dB	22.11 dB	23.91 dB	24.29 dB
Hall Monitor	33.24 dB	35.90 dB	33.89dB	36.65 dB	36.86 dB
Foreman	25.20 dB	31.41 dB	29.43 dB	32.02 dB	32.46 dB
